# Improved biosynthesis of heme in *Bacillus subtilis* through metabolic engineering assisted fed-batch fermentation

**DOI:** 10.1186/s12934-023-02077-3

**Published:** 2023-05-18

**Authors:** Shaomei Yang, Anlong Wang, Jiachang Li, Yunhang Shao, Fengjie Sun, Shucheng Li, Kai Cao, Hongliang Liu, Peng Xiong, Zhengquan Gao

**Affiliations:** 1 School of Life Sciences and Medicine, Shandong University of Technology, 266 Xincun West Road, Zibo, 255000 China; 2 School of Pharmacy, Binzhou Medical University, 346 Guanhai Road, Yantai, 256603 China; 3 School of Science and Technology, Georgia Gwinnett College, Lawrenceville, GA 30043 USA

**Keywords:** Heme biosynthesis, *Bacillus subtilis*, Endogenous C5 pathway, Uroporphyrinogen III synthesis pathway, Downstream synthesis pathway

## Abstract

**Background:**

Heme is an iron/porphyrin complex compound, widely used in the health care, food, and pharmaceutical industries. It is more advantageous and attractive to develop microbial cell factories to produce heme by fermentation, with lower production costs and environmentally more friendly procedures than those of the traditional extraction based on animal blood. In this study, *Bacillus subtilis*, a typical industrial model microorganism of food safety grade, was used for the first time as the host to synthesize heme.

**Results:**

The heme biosynthetic pathway was engineered as four modules, the endogenous C5 pathway, the heterologous C4 pathway, the uroporphyrinogen (urogen) III synthesis pathway, and the downstream synthesis pathway. Knockout of *hemX* encoding the negative effector of the concentration of HemA, overexpression of *hemA* encoding glutamyl-tRNA reductase, and knockout of *rocG* encoding the major glutamate dehydrogenase in the C5 pathway, resulted in an increase of 427% in heme production. Introduction of the heterologous C4 pathway showed a negligible effect on heme biosynthesis. Overexpression of *hemCDB*, which encoded hydroxymethylbilane synthase, urogen III synthase, and porphobilinogen synthase participating in the urogen III synthesis pathway, increased heme production by 39%. Knockouts of uroporphyrinogen methyltransferase gene *nasF* and both heme monooxygenase genes *hmoA* and *hmoB* in the downstream synthesis pathway increased heme production by 52%. The engineered *B. subtilis* produced 248.26 ± 6.97 mg/L of total heme with 221.83 ± 4.71 mg/L of extracellular heme during the fed-batch fermentation in 10 L fermenter.

**Conclusions:**

Strengthening endogenous C5 pathway, urogen III synthesis pathway and downstream synthesis pathway promoted the biosynthesis of heme in *B. subtilis*. The engineered *B. subtilis* strain has great potential as a microbial cell factory for efficient industrial heme production.

**Supplementary Information:**

The online version contains supplementary material available at 10.1186/s12934-023-02077-3.

## Background

Heme is an iron/porphyrin complex compound, essential for the survival of virtually all living organisms from bacteria, fungi, and yeast, through plants to animals [1]. Heme is a cofactor of many proteins and enzymes, and participates in various physiological and biochemical cellular processes. For example, hemoglobin is responsible for the transport/storage of oxygen, cytochromes promote electron transfer in the respiratory chains, and catalases/peroxidases have vital functions in oxidative stress detoxification [2, 3]. Since heme iron is easily absorbed and utilized by human cells without any side effects, it is commonly used as an iron supplement to treat anemia [4, 5]. Moreover, as a safe, natural pigment in food color enhancement and production, heme is also used to replace carcinogenic chromogen nitrite and other synthetic pigments [6]. Heme can also be used to prepare porphyrin derivatives, which are photosensitive and fluorescent, and used for diagnosis and treatment of diseases [7].

Current heme production is mostly performed by extraction from fresh pig blood, but there are significant limitations on the collection, transportation, and storage of the raw materials, resulting in high production costs and environmental pollution by the chemical reagents used in large quantities in the extraction process [8]. However, it is more advantageous and attractive to develop microbial cell factories to produce heme by means of synthetic biology, and it could effectively solve the problems associated with the traditional method. In microorganisms, the biosynthetic pathway of heme is divided into three modules, 5-aminolevulinate (ALA) synthesis module, uroporphyrinogen (urogen) III synthesis module and heme synthesis module. Depending on the species, the ALA synthesis module can have one of the parallel C5 and C4 pathways, and the heme synthesis module includes the protoporphyrin-dependent (PPD) pathway, the coproporphyrin-dependent (CPD) pathway, and the siroheme-dependent (SHD) pathway [3, 9].

In recent years, many researchers have used *Escherichia coli* as the host to build microbial cell factories for heme production [10, 11]. Zhao et al. constructed four expression plasmids to overexpress the endogenous C5, urogen III and PPD pathways, and heme exporter CcmABC, and knocked out phosphate acetyl transferase gene *pta*, lactate dehydrogenase gene *ldhA*, and heme-degrading enzyme gene *yfeX*. The engineered *E. coli* produced 239.2 mg/L of total heme with 151.4 mg/L of extracellular heme during fed-batch fermentation [12]. Furthermore, its heme production reached up to 1.03 g/L by increasing cell density, regular iron supplementation, and supply of excess feeding solution [13]. However, endotoxins secreted by *E. coli* precipitates an acute inflammatory response that often leads to shock and death [14, 15]. Therefore, heme produced by the engineered *E. coli* may not be used in the health care, food, and pharmaceutical industries. Therefore, it is necessary to find a safer host to produce heme. Ko et al. used *Corynebacterium glutamicum* as the host, and overexpressed the heterologous C4, the endogenous C5 and CPD pathways, the heme transporter HrtBA and the diphtheria toxin repressor protein DtxR, and defected heme-binding membrane proteins. The engineered *C. glutamicum* resulted in 309.18 mg/L of total heme with 242.95 mg/L of extracellular heme during fed-batch fermentation [16].

**Fig. 1:**
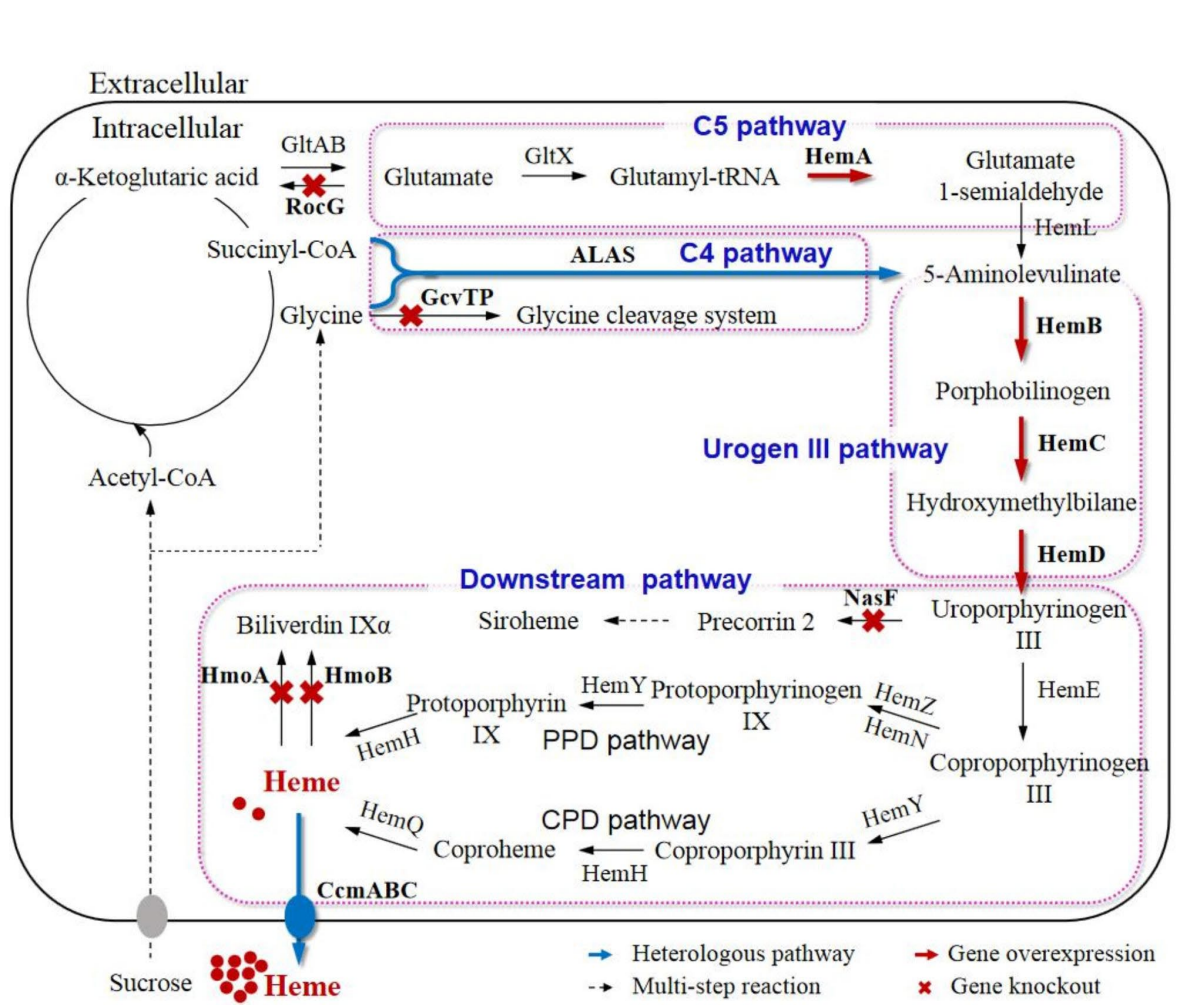
Schematic diagram of the heme biosynthetic pathway and overall engineering strategy in *B. subtilis*. GltA, glutamate synthase (large subunit); GltB, glutamate synthase (small subunit); GltX, glutamyl-tRNA synthetase; HemA, glutamyl-tRNA reductase; HemL, glutamate-1-semialdehyde aminotransferase; ALAS, 5-aminolevulinate synthase; HemB, porphobilinogen synthase; HemC, hydroxymethylbilane synthase; HemD, uroporphyrinogen (urogen) III synthase; HemE, urogen III decarboxylase; HemZ, coproporphyrinogen III oxidase; HemN, anaerobic coproporphyrinogen III oxidase; HemY, coproporphyrinogen oxidase; HemH, coproporphyrin ferrochelatase; HemQ, coproheme decarboxylase; CcmABC, heme exporter; RocG, glutamate dehydrogenase; GcvT, aminomethyltransferase (glycine cleavage system protein T); GcvPA, glycine decarboxylase subunit 1; GcvPB, glycine decarboxylase subunit 2; NasF, uroporphyrinogen methyltransferase; HmoA and HmoB, heme monooxygenase

*Bacillus subtilis* is an extensively used industrial microorganism, which is acceptable for food use and has been frequently subjected to metabolic engineering, because of its non-pathogenicity, strong ability to secrete extracellular proteins, and apparent absence of codon preference [17, 18]. Moreover, the genomic modification method for *B. subtilis* is very mature and convenient, however to the best of our knowledge, there is no report of heme production with engineered *B. subtilis*. In *B. subtilis*, ALA is synthesized from glutamate through the C5 pathway, which consists of three reactions catalyzed by enzymes GltX, HemA, and HemL, respectively. Subsequently, ALA is used to synthesize urogen III through the urogen III synthesis pathway, which is composed of three reactions catalyzed by enzymes HemB, HemC, and HemD, respectively, with urogen III ultimately decarboxylated to form coproporphyrinogen III (coprogen III) (Fig. 1). Coprogen III is converted into heme *via* either the PPD pathway (HemZ/HemN, HemY, and HemH), or the CPD pathway (HemY, HemH, and HemQ), and the CPD pathway is the main heme synthesis pathway in *B. subtilis* [19].

In this study, wild-type *B. subtilis* 168 was used as the microbial chassis for heme production for the first time. The heme biosynthetic pathway was engineered as four modules (Fig. 1), the endogenous C5 pathway, the heterologous C4 pathway, the urogen III synthesis pathway, and the downstream synthesis pathway. Each module was engineered by genomic editing to investigate the effect on heme synthesis and determine the key factors limiting heme production, thereby constructing high-producing strains. The final recombinant strain was subjected to fermentation optimization, by varying the carbon source and the composition of the feed solution, to further improve the heme yield. This study has provided a solid foundation for the further development of heme-producing cell factories and the industrial production of heme.

## Results

### Engineering the endogenous C5 pathway

**Fig. 2:**
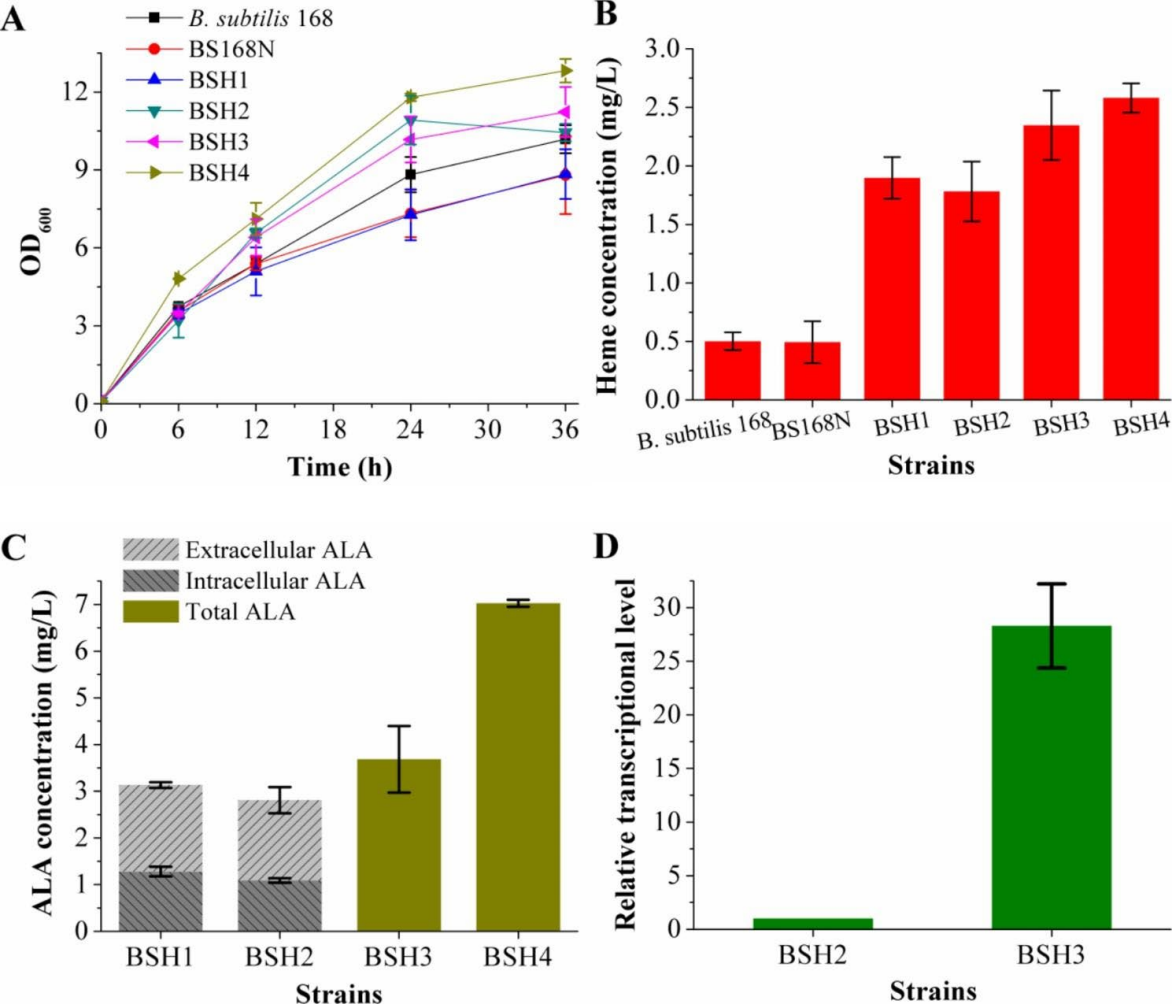
Growth curves, heme production, ALA production and gene transcriptional level of the recombinant strains constructed by engineering the endogenous C5 pathway. **(A)** Growth curves of wild-type *B. subtilis* 168, starting strain BS168N and recombinant strains BSH1, BSH2, BSH3, and BSH4. **(B)** Total heme production of *B. subtilis* 168, BS168N and recombinant strains after 36 h of fermentation. **(C)** Extracellular and intracellular ALA production of BSH1 and BSH2, total ALA production of BSH3 and BSH4 after 36 h of fermentation. **(D)** Transcriptional level of the *hemA* gene in BSH3, relative to that in BSH2, defined as 1

To facilitate the use of the marker-free method to modify the genome [20, 21], BS168N was used as the starting strain, with the neomycin resistance gene *neo*, regulated by the P_*ara*_ promoter and integrated into the *araR* site of the *B. subtilis* 168 genome [22]. The results showed that the introduction of the *neo* gene had little effect on bacterial growth and heme production (Fig. 2A and B). In *B. subtilis*, the membrane protein HemX decreases the steady-state cellular concentration of HemA protein by controlling its synthesis rate; the *hemX* gene is located in the *hemAXCDBL* operon, encoding the enzymes involved in urogen III biosynthesis from glutamate [23, 24]. HemA is the key enzyme in ALA biosynthesis *via* the C5 pathway in *B. subtilis* and ALA is an essential precursor for heme biosynthesis. The *hemX* gene was knocked out from the BS168N genome to obtain strain BSH1. It could produce 1.90 ± 0.18 mg/L heme after fermentation for 36 h, which were 287% higher than those of BS168N (Fig. 2B), suggesting that knockout of *hemX* markedly promoted heme biosynthesis.

BSH1 produced 3.13 ± 0.16 mg/L of ALA after 36 h of fermentation, of which 1.85 ± 0.06 mg/L was extracellular (Fig. 2C). Secretion of ALA is unfavorable for heme synthesis, but the ALA exporter of *B. subtilis* is unknown. Given that RhtA is the ALA exporter of *E. coli* [25], BLAST analysis was performed on the amino acid sequence of RhtA in the *B. subtilis* 168 database. There was high homology between YwfM in the EamA transporter family with RhtA. The *ywfM* gene was knocked out to obtain strain BSH2. However, knockout of *ywfM* resulted in 23% and 6% decreases in total ALA and heme production, respectively (Fig. 2C and B). Next, the strong constitutive promoter P_*43*_ [26] and the terminator of the *pyrE* gene were used to overexpress the *hemA* gene, obtaining recombinant strain BSH3. Its total ALA and heme production reached 3.68 ± 0.71 mg/L and 2.35 ± 0.30 mg/L, 53 and 32% higher, respectively, than that of BSH2 (Fig. 2C and B). In addition, RT-qPCR determined that mRNA transcription of *hemA* increased 27.3-fold (Fig. 2D), indicating that the use of P_*43*_ promoter to overexpress *hemA* was effective.

Glutamate is the direct precursor of ALA *via* the C5 pathway in *B. subtilis*. There are two genes encoding glutamate dehydrogenase (GlutDH) in *B. subtilis*, of which *rocG* encodes the major GlutDH [27], catalyzing the dehydrogenation of glutamate to generate α-ketoglutarate. Furthermore, RocG can bind directly to the transcription factor GltC to prevent its up-regulation of the glutamate synthase gene *gltAB*, thereby inhibiting glutamate biosynthesis [28]. Therefore, the *rocG* gene was knocked out to both reduce glutamate consumption and increase biosynthesis, which also improved the bacterial growth of the resulting recombinant strain, BSH4 (Fig. 2A). Total ALA concentration of BSH4 was 7.02 ± 0.08 mg/L, 91% higher than that of BSH3 (Fig. 2C), and its heme production was 2.58 ± 0.12 mg/L, 9.8% higher than that of BSH3 (Fig. 2B). These results indicated that knockout of *rocG* was beneficial for ALA and heme biosynthesis.

### Introduction of the heterologous C4 pathway

**Fig. 3:**
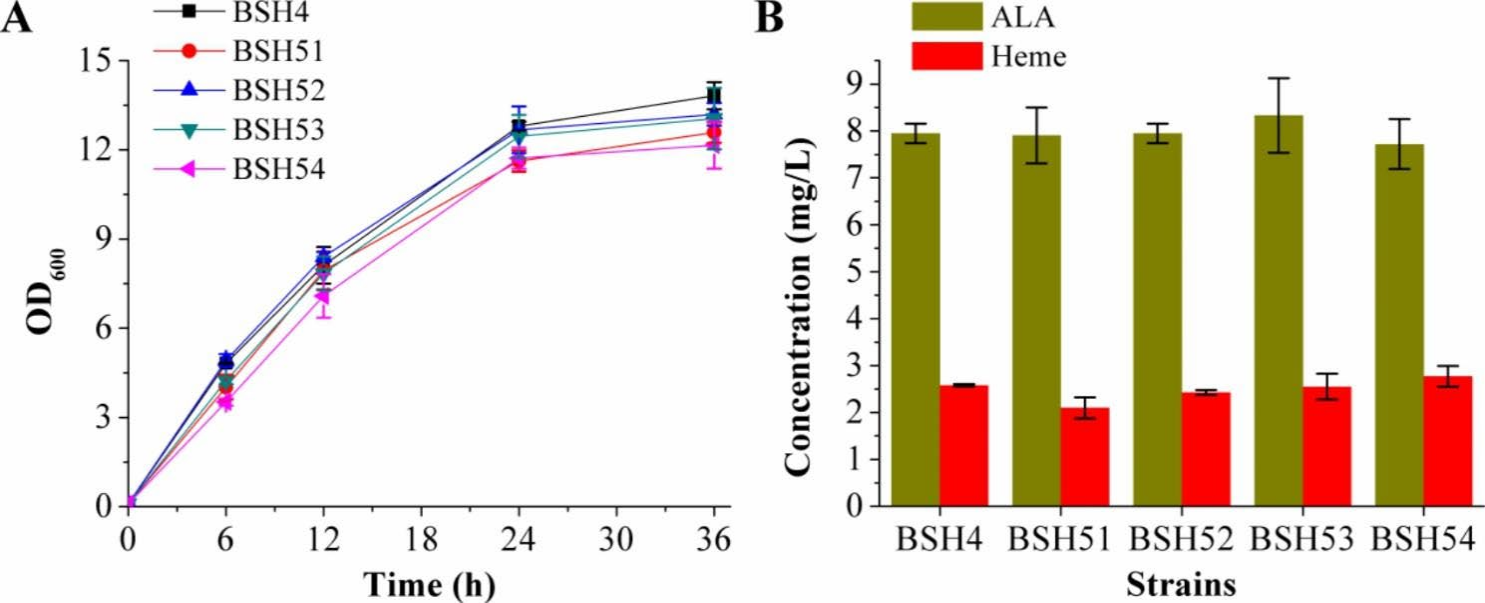
Growth curves, ALA and heme production of the recombinant strains constructed by introducing the heterologous C4 pathway. **(A)** Growth curves of the control strain BSH4 and the recombinant strains BSH51-BSH54. **(B)** Total ALA and heme production of these strains after 36 h of fermentation

**Fig. 4:**
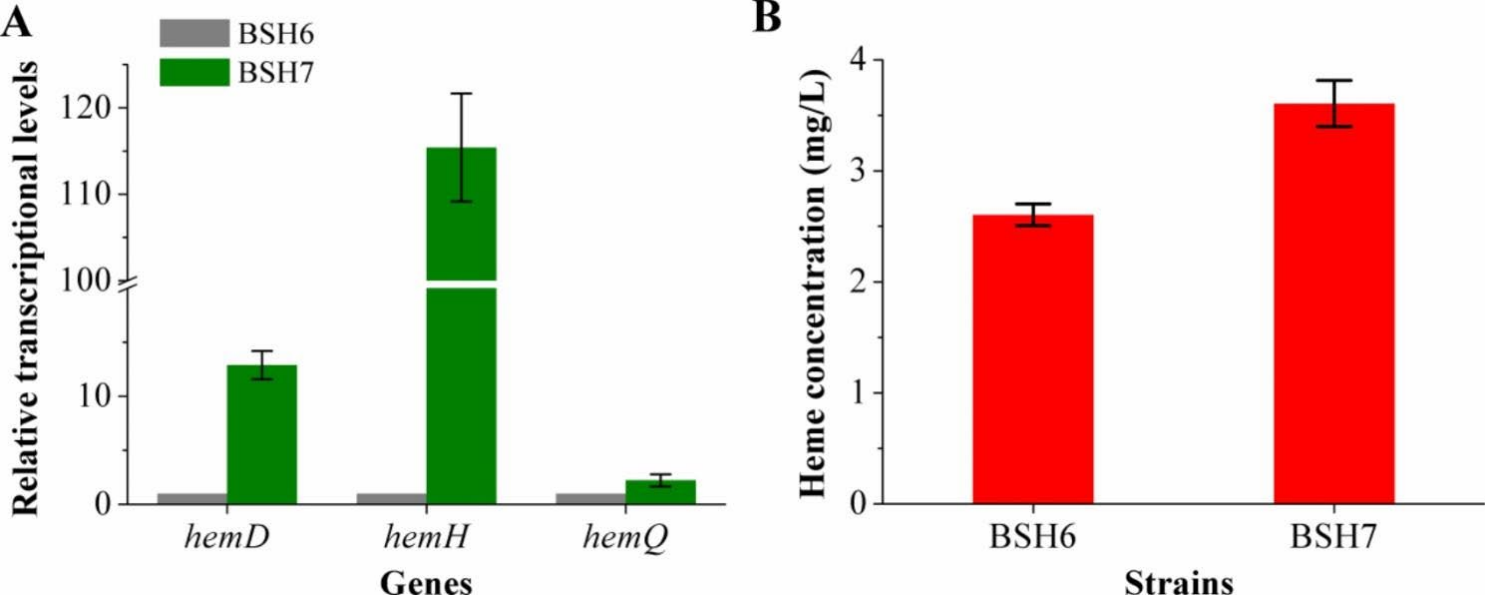
Relative transcriptional levels and heme production of the recombinant strains constructed by engineering the urogen III synthesis pathway. **(A)** Relative transcriptional levels of genes *hemD*, *hemH*, and *hemQ* in the recombinant strain BSH7, relative to that in BSH6, defined as 1. **(B)** Heme production of BSH6 and BSH7 after 36 h of fermentation

In mammals, fungi, and α-proteobacteria, ALA is synthesized via the C4 pathway, i.e., ALA synthetase (ALAS) catalyzes the condensation of succinyl-CoA and glycine to generate ALA with the release of both CO_2_ and coenzyme A [3, 29]. To explore the effect of introducing the heterologous C4 pathway on ALA and heme biosynthesis, the ALA synthase genes, *alaS*, from *Rhodopseudomonas palustris*, *R. capsulatus*, *Bradyrhizobium japonicum*, and *Agrobacterium tumefaciens* were codon optimized and synthesized, then expressed by insertion into the *rocG* locus on the genome of BSH3, regulated by P_*43*_ promoter, to obtain strains BSH51, BSH52, BSH53, and BSH54, respectively. The integrated expression of *alaS* from *R. palustri* and *(A) tumefaciens* slightly decreased the growth of recombinant strains compared with that of BSH4, whereas the growths of strain BSH52 overexpressing *alaS* from *R. capsulatus* and strain BSH53 overexpressing *alaS* from *(B) japonicum* were almost the same as that of BSH4 (Fig. 3A). After 36 h of fermentation, ALA concentration of the four recombinant strains were higher than that of BSH4, with *alaS* from *B. japonicum* (BSH53) producing the greatest increase (~ 4.8%) in ALA concentration (Fig. 3B). However, the introduction of heterogenous C4 pathway failed to significantly promote heme synthesis (Fig. 3B). In addition, a three-gene operon (*gcvT-gcvPA-gcvPB*) encodes the components of the glycine cleavage system [30], and glycine is one of the two precursors for the synthesis of ALA *via* the C4 pathway. The *gcvTP* operon in the genome of strain BSH53 was knocked out to obtain strain BSH6, which achieved the heme production of 2.60 ± 0.10 mg/L (Fig. 4B), showing no significant difference from that of strain BSH53 (2.55 ± 0.28 mg/L).

### Engineering the uroporphyrinogen III synthesis pathway

Eight molecules of ALA are needed to synthesize one molecule of urogen III, in a three-step pathway catalyzed by HemB, HemC and HemD; HemD (urogen III synthase) is responsible for cyclisation of the linear tetrapyrrole, hydroxymethylbilane [31]. Genes *hemC*, *hemD*, and *hemB* are closely linked within the *hemAXCDBL* operon in the *B. subtilis* genome. To promote the conversion of ALA to urogen III, the strong promoter P_*lapS*_ [32] was used to overexpress *hemCDB*, and integrate it into the *gcvTP* locus on the genome of BSH53, to obtain strain BSH7. Since *hemD* is located in the middle of genes *hemCDB*, its transcriptional level was measured to evaluate the effect of the promoter. RT-qPCR determined that mRNA transcriptional level of *hemD* increased 11.9-fold in BSH7 (Fig. 4A), indicating that the use of the P_*lapS*_ promoter to overexpress *hemCDB* was effective. Besides, transcription of gene *hemH*, in the middle of the *hemEHY* operon responsible for the CPD pathway [33], and gene *hemQ* in BSH7 was increased by 114.4-fold and 1.2-fold compared with BSH6, respectively (Fig. 4A). However, total heme produced by BSH7 reached 3.61 ± 0.21 mg/L after 36 h of fermentation, only 39% higher than that of the *gcvTP* knockout strain BSH6 (Fig. 4B).

### Engineering the downstream synthesis pathway

**Fig. 5:**
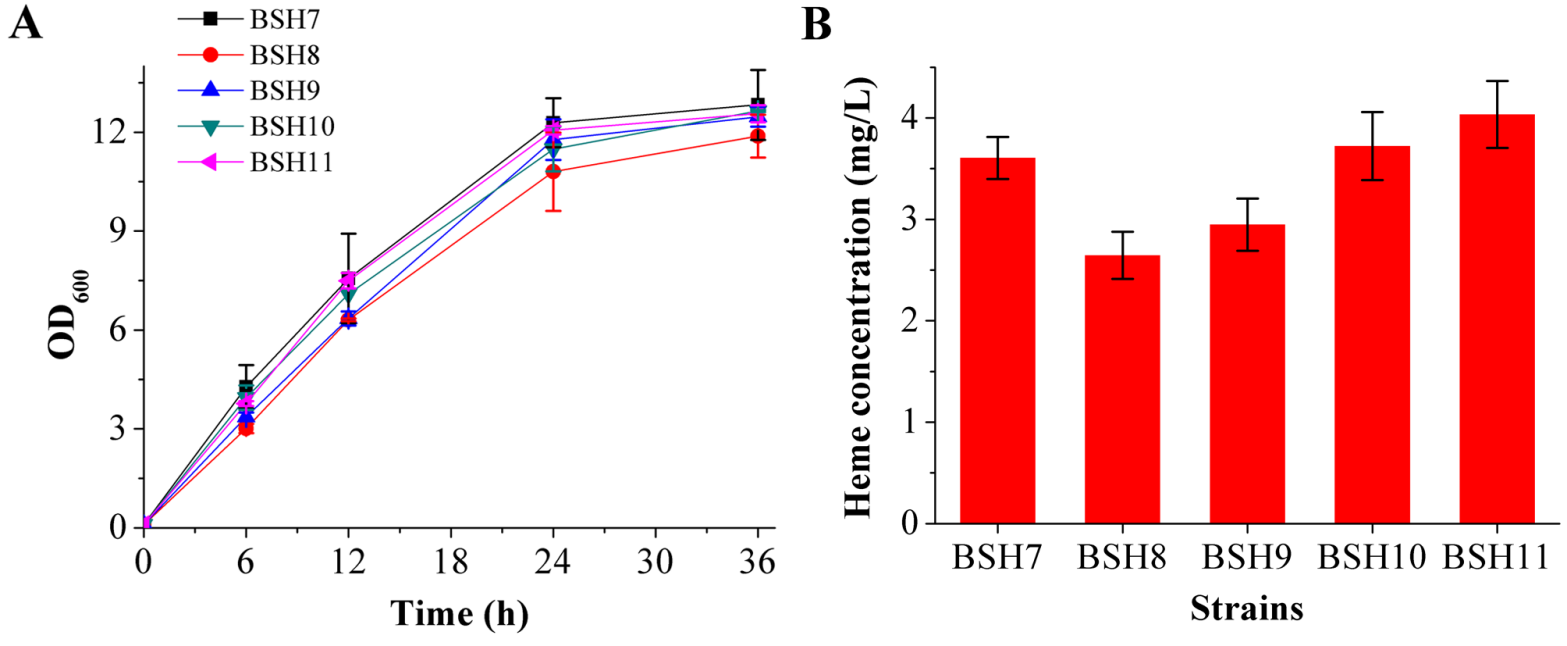
Growth curves and heme production of the recombinant strains constructed by engineering the downstream synthesis pathway of heme. **(A)** Growth curves of the control strain BSH7 and the recombinant strains BSH8-BSH11. **(B)** Heme production of BSH7-BSH11 after 36 h of fermentation

Heme is both hydrophobic and reactive, so “free” diffusion of heme through the cell membrane is not likely [34]; transport of heme may, therefore, be a key factor limiting its biosynthesis. However, little is known about the heme exporter in *B. subtilis*. Since the *ccmABC* genes from *E. coli* encode the heme exporter [12], which facilitates secretion of intracellular heme, recombinant strain BSH8 was constructed to express CcmABC. However, the growth of BSH8 was slightly lower than that of BSH7 **(Fig. 5A)**, and heme production decreased by 27% (Fig. 5B).

In addition, to being a key intermediate in heme biosynthesis, urogen III can also be converted by the methyltransferase NasF into precorrin-2 (Fig. 1), which is subsequently converted to siroheme, a cofactor for some enzymes [24]. To reduce the competitive depletion of urogen III, a *nasF* knockout strain (BSH9) was constructed, with heme production increased by 11% (Fig. 5B). There are two genes, i.e., *hmoA* (also known as *yetG*) and *hmoB* (also known as *yhgC*), encoding heme monooxygenase, which catalyzes the rate-limiting step in the heme degradation pathway of *B. subtilis* [35, 36]. To reduce the intracellular degradation of heme, a *hmoA* single knockout strain (BSH10), and the *hmoA*/*hmoB* double knockout strain (BSH11) were constructed. The growth of these two strains was slightly improved (Fig. 5A), and total heme production increased by 26 and 37% compared with that of BSH9, respectively, to 3.72 ± 0.34 mg/L and 4.03 ± 0.33 mg/L (Fig. 5B).

### Fed-batch fermentations

**Fig. 6:**
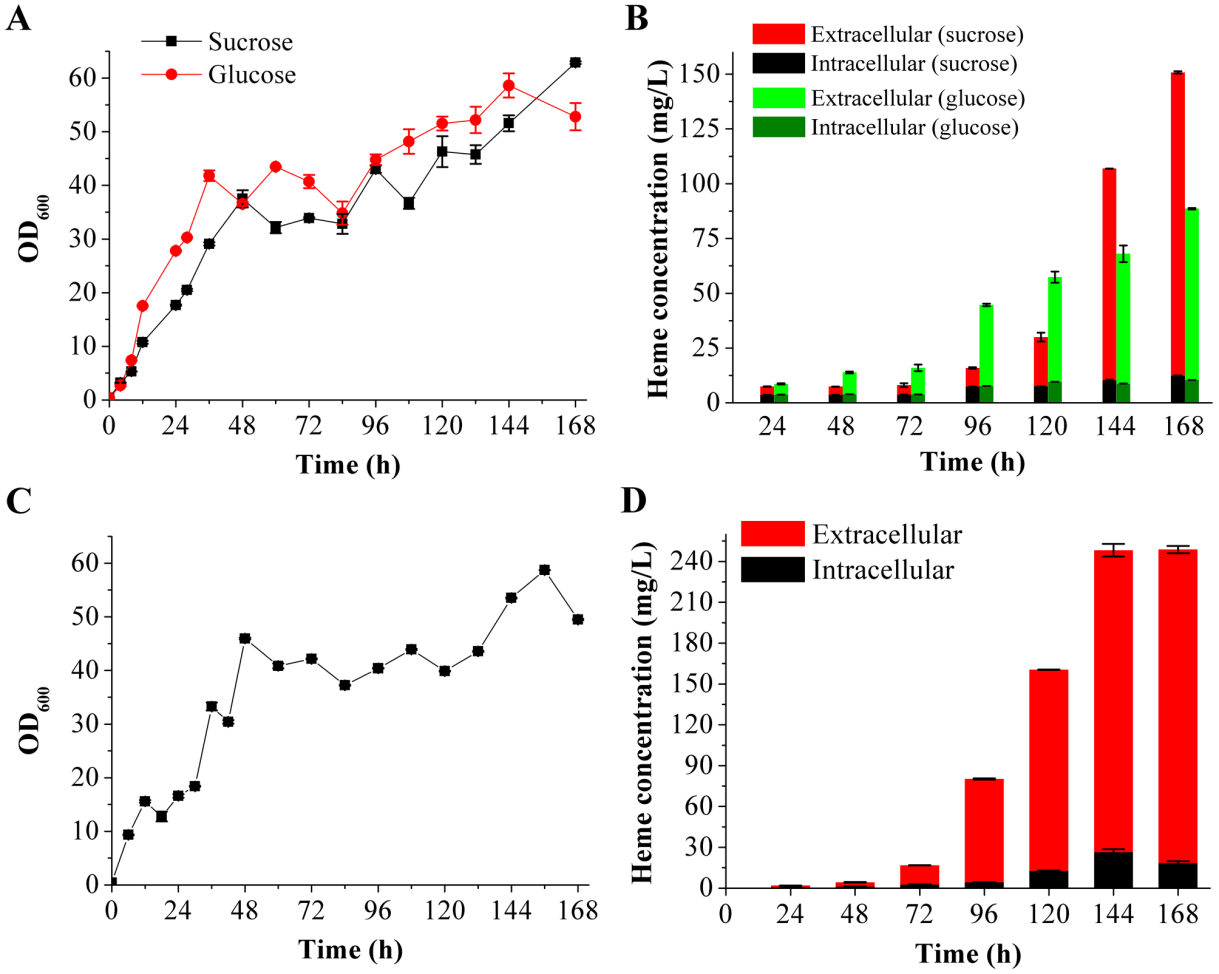
Growth curve and heme production of the final recombinant strain BSH11 during batch or fed-batch fermentations in fermenters. **(A)** Growth curves of BSH11 during fed-batch fermentation with sucrose or glucose as carbon sources in 2 L fermenters, respectively. **(B)** Extracellular and intracellular heme concentrations of BSH11 during fed-batch fermentation with sucrose or glucose as carbon sources in 2 L fermenters, respectively. **(C)** Growth curve of BSH11 during fed-batch fermentation in a 10 L fermenter. **(D)** Extracellular and intracellular heme concentrations of BSH11 during fed-batch fermentation in a 10 L fermenter

A fed-batch fermentation of BSH11 was performed in a 2 L fermenter with 400 g/L sucrose and 40 g/L tryptone as the feed solution, resulting in a longer stationary phase for BSH11 (Fig. 6A). Total heme content at the 144th and 168th hours of fermentation was 106.88 ± 0.31 mg/L and 150.78 ± 0.59 mg/L, respectively, and those of extracellular heme were 96.55 ± 0.01 mg/L and 138.41 ± 0.51 mg/L, accounting for 90% and 92% of the total, respectively (Fig. 6B). Since the previous studies used glucose as the carbon source [12, 16], sucrose in both the fermentation medium and the feed solution was replaced with glucose in this study. The growth of BSH11 was slightly higher with glucose than sucrose as carbon source (Fig. 6A); total heme production was markedly higher with glucose for the first 120 h of fermentation, but markedly higher with sucrose after 120 h (Fig. 6B). Extracellular heme content at the 144th and 168th hours of fermentation was 58.66 ± 3.76 mg/L and 77.40 ± 0.31 mg/L, respectively (Fig. 6B), 39 and 44% lower than those with sucrose as carbon source, respectively. Finally, fed-batch fermentation of BSH11 was performed in a 10 L fermenter supplemented with 400 g/L glucose, 40 g/L tryptone and 7.5 mg/L FeSO_4_·7H_2_O for the first 96 h of fermentation, then sucrose replaced glucose in the feed solution until the end of fermentation. The growth of BSH11 was not significantly different from that in the 2 L fermenter (Fig. 6C), however, total heme concentration at the 144th hour of fermentation reached 248.26 ± 6.97 mg/L (Fig. 6D). Extracellular heme concentration was 221.83 ± 4.71 mg/L, accounting for 89% of the total.

## Discussion

ALA is the direct precursor of heme biosynthesis, which is synthesized by glutamate *via* C5 pathway in *B. subtilis*. HemA is the key enzyme of C5 pathway, and its intracellular concentration is regulated by HemX [23, 24]. Knockout of *hemX* increased the heme production by 287%, indicating that the regulation of HemA was released, thus promoting the heme biosynthesis. Since 59% of the ALA produced by the *hemX* knockout strain was secreted outside the cell, if the ALA exporter could be found, more ALA would flow into the heme synthesis pathway by defective its expression. Comparative analysis of the amino acid sequence of ALA exporter RhtA of *E. coli* [25] based on the database of *B. subtilis* revealed that the amino acid sequence of unknown protein YwfM showed the highest homology with that of RhtA. However, knockout of *ywfM* showed no positive effect on the synthesis of ALA and heme. It was speculated that there was more than one gene encoding ALA exporter in *B. subtilis*, and the effect of knocking out *ywfM* might be compensated by other proteins. Moreover, overexpression of *hemA* increased the total production of ALA and heme by 53% and 32%, respectively, further indicating that HemA was the key enzyme, and its expression level was increased, which promoted the ALA synthesis, and thus increased the heme production. In addition, knockout of *rocG* increased the total production of ALA and heme by 91% and 9.8%, respectively. Although the ALA synthesis was significantly increased, the failure to prevent its outflow resulted in a relatively small amount of ALA flowing into the heme synthesis pathway, ultimately causing a small increase in heme production.

The expression of four different sources of ALAS had no significant positive effect on ALA and heme synthesis. And knockout of *gcvTP* has no obvious effect on heme synthesis. It suggested that ALA synthesized by increasing the metabolic flux of the endogenous C5 pathway was sufficient for heme synthesis. It might be that the metabolic flux of urogen III synthesis pathway or heme downstream synthesis pathway was low, which limited the positive effect of C4 pathway on heme synthesis. Therefore, *hemCDB* was overexpressed to strengthen the urogen III synthesis pathway, and the heme production increased by 39%. Moreover, the transcriptional levels of both *hemH* and *hemD* in the CPD pathway were also increased, probably due to the increased synthesis of urogen III, promoting its conversion to heme, i.e., increasing the expression level of the main synthesis pathway genes. *hemH* encodes coproporphyrin ferrochelatase and is located in the middle of the *hemEHY* operon. It was inferred that the native promoter of this operon might be activated by urogen III or other factors, resulting in a significant increase in its transcriptional level. It might be that the toxicity of accumulated intracellular heme to the cells resulted in the intensification of urogen III synthesis pathway having a relatively insignificant effect. Disappointingly, the expression of the heme exporter CcmABC from *E. coli* reduced heme production by 27%. Since the heme synthesis and secretion of recombinant *E. coli* constructed by overexpressing *ccmABC* did not significantly increase in flask culture, these were significantly increased during fed-batch fermentation in a stirred fermenter [12]. Therefore, it might be that flask fermentation in this study limited the dissolved oxygen (DO) content sufficiently to impair fermentation performance. In addition, knockouts of *nasF*, *hmoA* and *hmoB* increased heme production by 52%, indicating that blocking the competitive depletion of urogen III and the intracellular degradation of heme were both beneficial to improve the heme production. Since the overexpression of *hemCDB* increased the transcription of both *hemH* and *hemQ* in the CPD pathway, there was no evident increase in heme biosynthesis, suggesting that the heme production might be limited by fermentation scale because fermentation in the bioreactor could control DO and pH, increase stirring speed, supplement culture medium, etc.

The heme yield of the final recombinant strain BSH11 was up to 150.78 ± 0.59 mg/L after fed-batch fermentation in 2 L fermenter, and its secretion ratio was 92%. When sucrose was replaced with glucose of the same concentration, the heme yield reached 87.77 ± 0.32 mg/L, with the secretion ratio of 88%. Overall, both glucose and sucrose as fermentation carbon sources increased heme biosynthesis; glucose produced more heme in the early and middle stages of fermentation, whereas sucrose produced more heme towards the end of fermentation. Finally, we conducted the fed-batch fermentation in a 10 L fermenter, with glucose as the carbon source in the early and middle stages of fermentation, and then changing it to sucrose. Since Fe^2+^ is also an important factor in heme biosynthesis, 7.5 mg/L FeSO_4_·7H_2_O had been supplemented. The maximum heme yield reached 248.26 ± 6.97 mg/L, and its secretion ratio was 89%. The above results show that the heme secretion ratio of *B. subtilis* BSH11 had been fluctuating between 88 and 92%, and the larger-scale fermentation and supplementation of ferrous ions further improved the heme yield. In the future, it is necessary to further optimize the composition of feeding medium and increase cell density, which will further promote the heme biosynthesis. This study used constitutive promoters to overexpress key genes and integrate them into the genome, instead of inducible promoters and expression plasmids, which enhanced the stability of recombinant strains and would reduce the fermentation cost of industrial production. Therefore, the engineered *B. subtilis* strain BSH11 should be a promising microbial cell factory for industrial heme production.

## Materials and methods

### Microorganisms and culture conditions

All recombinant *B. subtilis* strains were derived from the strain BS168N [22] donated by Tianjin University (Table 1). Strains were grown in Luria-Bertani (LB) liquid medium (containing 10 g/L tryptone, 5 g/L yeast extract, and 10 g/L NaCl). Solid medium was obtained by adding 15 g/L agar to the liquid medium. As required, antibiotic (16 µg/mL neomycin or 8 µg/mL chloramphenicol) was added to the growth media for the selection of *B. subtilis*.

**Table 1:** Strains and plasmids used in this study

Name	Relevant strain and genotype	Sources
***Strains***		
*B. subtilis* 168	*trpC2*	Donated by Tianjin University [22]
BS168N	*trpC2*, Δ*araR*::P_*ara*_-*neo*	Donated by Tianjin University [22]
BS168NUm	*trpC2*, Δ*araR*::P_*ara*_-*neo*, Δ*upp*::*cat-araR*	Donated by Tianjin University [22]
*E. coli* DH5α	F^–^, Φ80*lacZ*ΔM15, Δ(*lacZYA-argF*), *U*16, *recA*1, *endA*1, *hsdR*17 (rK^–^, mK^+^), *phoA*, *supE*44, λ^–^, *thi*1, *gyrA*96, *relA*1	Laboratory stock
BSH1	BS168N, Δ*hemX*	This study
BSH1Ym	BS168N, Δ*hemX*, Δ*ywfM*::D-*cat-araR*	This study
BSH2	BS168N, Δ*hemX*, Δ*ywfM*	This study
BSH3	BS168N, Δ*hemX*, Δ*ywfM*::P_*43*_-*hemA*	This study
BSH4	BS168N, Δ*hemX*, Δ*ywfM*::P_*43*_-*hemA*, Δ*rocG*	This study
BSH51	BS168N, Δ*hemX*, Δ*ywfM*::P_*43*_-*hemA*, Δ*rocG*::P_*43*_-*ALAS*_*Rp*_	This study
BSH52	BS168N, Δ*hemX*, Δ*ywfM*::P_*43*_-*hemA*, Δ*rocG*::P_*43*_-*ALAS*_*Rc*_	This study
BSH53	BS168N, Δ*hemX*, Δ*ywfM*::P_*43*_-*hemA*, Δ*rocG*::P_*43*_-*ALAS*_*Bj*_	This study
BSH54	BS168N, Δ*hemX*, Δ*ywfM*::P_*43*_-*hemA*, Δ*rocG*::P_*43*_-*ALAS*_*At*_	This study
BSH6	BSH53, Δ*gcvTP*	This study
BSH7	BSH53, Δ*gcvTP*::P_*lapS*_-*hemCDB*	This study
BSH8	BSH7, Δ*ywjI*::P_*lapS*_-*ccmABC*_*Ec*_	This study
BSH9	BSH7, Δ*ywjI*::P_*lapS*_-*ccmABC*_*Ec*_, Δ*nasF*	This study
BSH10	BSH7, Δ*ywjI*::P_*lapS*_-*ccmABC*_*Ec*_, Δ*nasF*, Δ*hmoA*	This study
BSH11	BSH7, Δ*ywjI*::P_*lapS*_-*ccmABC*_*Ec*_, Δ*nasF*, Δ*hmoA*, Δ*hmoB*	This study
***Plasmids***		
pUC57-1.8k-1	Amp^R^, containing the *alaS* gene from *R. palustris*, synthesized by Genecreate Biotech	This work
pUC57-1.8k-2	Amp^R^, containing the *alaS* gene from *R. capsulatus*, synthesized by Genecreate Biotech	This work
pUC57-1.8k-3	Amp^R^, containing the *alaS* gene from *B. japonicum*, synthesized by Genecreate Biotech	This work
pUC57-1.8k-4	Amp^R^, containing the *alaS* gene from *A. tumefaciens*, synthesized by Genecreate Biotech	This work
pUC57-1.8k-P1	Amp^R^, containing promoter P_*lapS*_	Donated by Tianjin University [22]

Amp^R^, ampicillin resistance.

### DNA manipulation techniques

Genomic DNA was extracted from *B. subtilis* using the TIANamp Bacteria DNA Kit (Tiangen, Beijing, China). The primers were synthesized by GenScript Biotech (Nanjing, China), then DNA sequenced by Genewiz Biotech (Suzhou, China). The primers used in this study are listed in Table S1. The *alaS* genes from *Rhodopseudomonas palustris* (GenBank accession AFU07636), *Rhodopseudomonas capsulatus* (GenBank accession WP_013067171), *Bradyrhizobium japonicum* (GenBank accession ABD39319), and *Agrobacterium tumefaciens* (GenBank accession AAR83718) were synthesized separately by Genecreate Biotech (Wuhan, China). DNA polymerases, 2 × Phanta Flash Master Mix (Dye Plus) and 2 × Taq Master Mix (Dye Plus) (Vazyme Biotech, Nanjing, China) were used, following the manufacturer’s instructions. The overlap extension by polymerase chain reaction (OE-PCR) was carried out as described previously [37]. The marker-free gene modification was based on a previous report [20], and used for gene knockout and overexpression. The transformation of *B. subtilis* was performed by using competent cells, as described previously [38].

### Gene knockout

The knockout processes used for all target genes were essentially the same as that for the *hemX* gene (**Supporting Methods**), as follows. Fragments of 1,065 bp U_hemX_ covering the nucleotide sequences of gene *hemA*, 1,059 bp D_hemX_ covering the nucleotide sequences of *hemC*, and 752 bp G_hemX_ covering the nucleotide sequences of *hemX*, were amplified from the *B. subtilis* 168 genome, using the primer pairs hemX-U1/hemX-U2, hemX-D1q/hemX-D2, and hemX-G1q/hemX-G2, respectively. The 2,136 bp CR (*cat*-*araR*) fragment was amplified from BS168NUm using the primer pair hemX-CR1q/hemX-CR2. These four PCR fragments were then ligated in the order of U-D-CR-G by splicing with two rounds of OE-PCR, using the primer pairs hemX-U1/hemX-D2 and hemX-U1/hemX-G2, respectively. Finally, the UDCRG fragment was used to transform into the competent cells of BS168NU. The recombinant strain BSH1 was obtained by the two-step screening process, as described previously [20]. DNA sequencing was performed using the primers CX-hemX-1, CX-hemX-2, and CX-hemX-3.

### Gene overexpression

Overexpression of all target genes was essentially the same as for the *hemA* gene (**Supporting Methods**), as follows. Fragments of 1,245 bp U_hemA_, 267 bp P_hemA_ (containing the sequence of the constitutive promoter P_*43*_), 1,408 bp A_hemA_ (containing the gene sequence of *hemA*), and 259 bp T_hemA_ (containing the terminator sequence of *pyrE*) were amplified from the *B. subtilis* 168 genome, using the primer pairs ywfM-U1/ywfM-U2, ywfM-hemA-P1q/ywfM-hemA-P2, ywfM-hemA-1q/ywfM-hemA − 2 and ywfM-hemA-T1q/ywfM-hemA-T2, respectively. The 4,030 bp DCRG fragment was amplified from BSH1Ym using the primers ywfM-hemA-D1q/ywfM-hemA-G2. These five PCR fragments were then ligated in the order of U-P-A-T-DCRG, by splicing with two rounds of OE-PCR using the primer pairs ywfM-hemA-P1q/ywfM-hemA-T2 and ywfM-U1/ywfM-hemA-G2. Finally, competent cells of BSH1 were transformed using the UPATDCRG fragment yielding strain BSH3 with the successful transformation verified by a two-step screening process. DNA sequencing was performed by using the primers CX-P_*43*_-1 and ywfM-hemA-T2.

### Flask fermentation

Recombinant strains were streaked on LB solid medium and cultured overnight at 37 °C, then a single colony was picked out and transferred into a test tube containing LB liquid medium (5 mL), then cultured at 37 °C with shaking at 200 rpm, for 10 − 12 h. Finally, the seed culture (2%) was transferred to a 250 mL flask with fermentation medium (30 mL, containing 40 g/L sucrose, 4 g/L tryptone, 15 g/L (NH_4_)_2_SO_4_, 5 g/L KH_2_PO_4_, 15 g/L Na_2_HPO_4_·12H_2_O, 0.5 g/L MgSO_4_·7H_2_O, and 7.5 mg/L FeSO_4_·7H_2_O) and incubated for 36 h at 37 °C with shaking at 220 rpm. During the fermentation, samples were taken every 12 h and the absorbance was measured at 600 nm.

### Analytical methods

ALA standard was from Sigma-Aldrich (St. Louis, MO, USA). The ALA extraction and analysis methods were as described previously, with some modifications[39]. Sodium acetate buffer (125 µL, 8.2 g/L sodium acetate and 57 mL/L acetic acid) and acetylacetone (62.5 µL) were added to a centrifuge tube containing fermentation supernatant (500 µL), then heated at 100 °C for 15 min. After cooling, Modified Ehrlich’s reagent was added (440 µL, 1 g of *p*-dimethylaminobenzaldehyde, 5 mL of 70% perchloric acid, and 5 mL of distilled water added to 30 mL of acetic acid, then the volume adjusted to 50 mL with acetic acid) and left to stand at room temperature for 20 min. The sample was centrifuged at 12,000 rpm for 5 min, the supernatant retained and added to a white 96-well plate to measure the absorbance at 554 nm, with a microplate reader (Thermo, USA). Fermentation medium (0.5 mL) was used as the blank control, using the same treatment. The ALA concentration of the sample was calculated from a standard curve.

The heme standard was from Sigma-Aldrich. The cells were harvested by centrifugation and the supernatant assayed for extracellular heme. The cell pellet was resuspended in NaOH (0.1 M), then sonicated for 10 min in an ice bath. After a further centrifugation, the supernatants were used to determine intracellular heme. Heme was assayed on the reverse-phase high-performance liquid chromatography (HPLC) system (1260 Infinity II, Agilent, Santa Clara, CA) equipped with a ZORBAX SB-C18 column (250 mm × 4.6 mm, 5 μm, Agilent) and a UV-detector (1260 VWD, Agilent), set at 400 nm. The column temperature was set at 40 °C. The mobile phases were: 0.1% aqueous trifluoroacetic acid (A) and methanol (B); a gradient elution was performed at 0.4 mL/min as follows: 0 − 1 min, 30% B; 1 − 20 min, from 30% B to 100%B; 20 − 21 min, from 100% B to 30% B; and 21 − 35 min, 30% B [12].

### RT-qPCR

Determination of the transcriptional level of the *hemA* gene. A total of 1 mL of culture from strains BSH2 and BSH3 after 24 h of fermentation was collected, respectively, with the total RNAs extracted using a FastPure Cell/Tissue Total RNA Isolation Kit V2 (Vazyme). The RNA solutions were appropriately diluted and then measured the A_260_/A_280_ ratio using NanoDrop 2000 to calculate the content of RNA. The reverse transcription was performed using the HiScript III RT SuperMix for qPCR (+ gDNA wiper) (Vazyme) to obtain the cDNA libraries. Finally, the cDNAs of strains BSH2 and BSH3 were added to the PCR tube as templates using primers RT-ccpA-1/RT-ccpA-2 and RT-hemA-1/RT-hemA-2 (Table S1), respectively. The ChamQ Universal SYBR qPCR Master Mix (Vazyme) was added to the reaction system. RT-qPCR was carried out on a LightCycler 480 (Roche, Germany) to collect the fluorescence signal Ct based on the formula ΔΔCt = [(Ct_(target, test)_ – Ct_(ref, test)_) – (Ct_(target, calibrator)_ – Ct_(ref, calibrator)_], where “target” was the gene *hemA*, “test” was the sample BSH3, “ref” was the internal reference gene *ccpA*, and “calibrator” was the control sample BSH2. The relative transcriptional level of the gene *hemA* was calculated based on 2^–ΔΔCt^ (three parallel).

Determination of the transcriptional levels of genes *hemD*, *hemH*, and *hemQ*. A total of 1 mL of culture from strains BSH6 and BSH7 after 24 h of fermentation were collected, respectively, and total RNAs were extracted with a FastPure Cell/Tissue Total RNA Isolation Kit V2. The RNA contents were calculated, and the RNA was reverse-transcribed to cDNA using the HiScript III RT SuperMix for qPCR (+ gDNA wiper) by following the manufacturer’s instructions. RT-qPCR was carried out with the LightCycler 480 using the ChamQ Universal SYBR qPCR Master Mix to collect the fluorescence signal Ct based on the formula ΔΔCt = [(Ct_(target, test)_ – Ct_(ref, test)_) – (Ct_(target, calibrator)_ – Ct_(ref, calibrator)_], where “target” was genes *hemD*, *hemH*, and *hemQ*, respectively, “test” was the sample BSH7, “ref” was the internal reference gene *ccpA*, and “calibrator” was the control sample BSH6. The relative transcriptional levels of genes *hemD*, *hemH*, and *hemQ* were calculated based on 2^–ΔΔCt^, respectively (three parallel). [40].

### Fed-batch fermentations

Seed culture (100 mL) was inoculated into the 2 L stirred fermenter (BLBIO-2GC, Shanghai, China) with fresh fermentation medium (900 mL), and fermentation was carried out at 37 °C, with the pH maintained at 7.0 using NaOH (1 mol/L). Aeration was provided at 2 − 5 L/min, with agitation at 300 − 1,000 rpm. The feed solution containing 400 g/L sucrose and 40 g/L tryptone was added automatically to maintain the DO at 40%. When glucose was used as the carbon source for fermentation, the feed solution consisted of 400 g/L glucose and 40 g/L tryptone. For fed-batch fermentation in a 10 L fermenter (BLBIO-10SJ), seed culture (400 mL) was inoculated into the fermenter containing fermentation medium (3.6 L). The air flow rate was 15 L/min, with agitation at 250 − 800 rpm. During the first 96 h of fermentation, the feed solution consisted of 400 g/L glucose, 40 g/L tryptone and 7.5 mg/L FeSO_4_·7H_2_O; after 96 h, the glucose was replaced by sucrose.The feed solution was added automatically to maintain the DO at 40%.

## Electronic supplementary material

Below is the link to the electronic supplementary material.

**Additional file 1**: **Table S1.** Primers and their sequences used in this study. **Fig. S1** Color changes of the fermentation medium during fed-batch fermentation in a 2 L fermenter: (A) BSH11 fermented with sucrose as carbon source in both the fermentation medium and the feed solution for 7 days. (B) BSH11 fermented with glucose as carbon source for 7 days. **Supporting Methods** The construction processes of other different strains, i.e., knockout strains of *ywfM*, *rocG*, *gcvTP*, *nasF*, *hmoA*, and *hmoB*, and overexpression strains of *hemCDB*, and *ccmABC*.

## Data Availability

All data generated or analyzed during this study are included in this article and its additional information file.

## Abbreviations

ALA: *5-Aminolevulinate*
urogen: *Uroporphyrinogen*
PPD: *Protoporphyrin-dependent*
CPD: *Coproporphyrin-dependent*
SHD: *Siroheme-dependent*
