# Supplementary material for: Improved biosynthesis of heme in Bacillus subtilis through metabolic engineering assisted fed-batch fermentation
Source: Microb Cell Fact. 2023 May 18;22:102. doi: 10.1186/s12934-023-02077-3 (PMC10193802; doi:10.1186/s12934-023-02077-3)
Supplement: Supplementary file 1 — Additional file 1: Table S1. Primers and their sequences used in this study. Fig. S1 Color changes of the fermentation medium during fed-batch fermentation in a 2 L fermenter: (A) BSH11 fermented with sucrose as carbon source in both the fermentation medium and the feed solution for 7 days. (B) BSH11 fermented with glucose as carbon source for 7 days. Supporting Methods The construction processes of other different strains, i.e., knockout strains of ywfM, rocG, gcvTP, nasF, hmoA, and hmoB, and overexpression strains of hemCDB, and ccmABC. [file 12934_2023_2077_MOESM1_ESM.doc]

**Supporting Information**

**Improved biosynthesis of heme in *Bacillus subtilis* through metabolic engineering assisted fed-batch fermentation**

Shaomei Yang^1^, Anlong Wang^1^, Jiachang Li^1^, Yunhang Shao^1^, Fengjie Sun^3^, Shucheng Li^1^, Kai Cao^1^, Hongliang Liu^1^, Peng Xiong^1*^, Zhengquan Gao^2*^

^1^School of Life Sciences and Medicine, Shandong University of Technology, 266 Xincun West Road, Zibo 255000, China

^2^School of Pharmacy, Binzhou Medical University, 346 Guanhai Road, Yantai 256603, China

^3^School of Science and Technology, Georgia Gwinnett College, Lawrenceville, Georgia 30043, USA

**Supporting Table & Table legend**

**Table S1. Primers and their sequences used in this study.**

| **Primer** | **Sequence (5'→3')** |
| --- | --- |
| **Gene knockout** | |
| hemX-U1 | CCTGCGGACTTGATTCTAT |
| hemX-U2 | TCAACTTTCACTCACTTACAA |
| hemX-D1q | CTTGTAAGTGAGTGAAAGTTGAGCCTGTCGCAATTCCATT |
| hemX-D2 | CTGCCTGTGCCTTATTCC |
| hemX-CR1q | CGGAATAAGGCACAGGCAGGCACCCATTAGTTCAACAAA |
| hemX-CR2 | CTCACCAACATCTATACCAAC |
| hemX-G1q | CGTTGGTATAGATGTTGGTGAGGCAATGGCAAGACTTAATGA |
| hemX-G2 | CAAGCCGTATTCCACAGT |
| CX-hemX-1 | AACATCATTGTCTCAACCTG |
| CX-hemX-2 | ATCTGGAAGGCATTGTAGAA |
| CX-hemX-3 | GTTCCGCAACATCATTCG |
| ywfM-U1 | AGCAGCAATCAAGGTGTAA |
| ywfM-U2 | GTCGTCGTTATCTGTATGTAAG |
| ywfM-D1q | TCTTACATACAGATAACGACGACGGCAGCACACAAGTCTTA |
| ywfM-D2 | TTAGTGGCTTGGCAATCAT |
| ywfM-CR1q | GATGATTGCCAAGCCACTAACTTCAACTAAAGGACCCATTAG |
| ywfM-CR2 | TTATTCATTCAGTTTTCGTG |
| ywfM-G1q | GCACGAAAACTGAATGAATAATTTGGAGGGTTGAGGTTAT |
| ywfM-G2 | AGGAAGGCGAAAGCATTT |
| rocG-U1 | CGCCGCTGACAATTATCT |
| rocG-U2q | CTCAAATTAGACCCATCCGCAGTAGCCTGTGAACACTT |
| rocG-D1 | GCGGATGGGTCTAATTTGA |
| rocG-D2 | GTCTTCGGATGCTCAACTA |
| rocG-CR1q | TTAGTTGAGCATCCGAAGACGCACCCATTAGTTGAACAAA |
| rocG-CR2 | CACACCAACATCTATACCAAC |
| rocG-G1q | CGTTGGTATAGATGTTGGTGTGTCAGAAGTTAATGAAGAGGAAG |
| rocG-G2 | TTATGTGTTGCCGCTGTT |
| gcvTP-U1 | GCGGTGTCTATTGATGAGA |
| gcvTP-U2 | GTATAGCCAGTGCGTGAA |
| gcvTP-D1q | TTTCACGCACTGGCTATACTACGGCAACTTTGGCATT |
| gcvTP-D2 | ACGGCTCTATGACCTTGT |
| gcvTP-CR1q | AACAAGGTCATAGAGCCGTAAGCAGCCATTAGTTCAACA |
| gcvTP-CR2 | CACACCAACATCCATTCCAAC |
| gcvTP-G1q | GTTGGAATGGATGTTGGTGTGCTGTTGTAGATGTAGAGATACG |
| gcvTP-G2 | GTGCCTGGTGCTGATTAC |
| nasF-U1 | AACATACTCAGTCGTTCCTC |
| nasF-U2 | TGTCATCCTTCCGTCACT |
| nasF-D1q | CAGTGACGGAAGGATGACAGCATAGGCGATGACTCAG |
| nasF-D2 | TGAAGGCGTAATGATGGAAT |
| nasF-CR1q | TATTCCATCATTACGCCTTCAGCACCCATTAGTTCAACAAA |
| nasF-G1q | CGTTGGTATAGATGTTGGTGAGTGAAGGAAGCAGATGTGATT |
| nasF-G2 | GAATGGCGGTGACAAGAA |
| hmoA-U1 | ATTCACGGTCTGTCTCCT |
| hmoA-U2 | TGTCTCTCTCCAATCATTCT |
| hmoA-D1q | GAGAATGATTGGAGAGAGACAGTTCAACCGCAACACTTAAAT |
| hmoA-D2 | TCACTGGATGATATGGAAGTC |
| hmoA-CR1q | CGACTTCCATATCATCCAGTGAGCACCCATTAGTTCAACAAA |
| hmoA-G1q | CGTTGGTATAGATGTTGGTGTGGGTGTTGAAGAAGTTGTTGT |
| hmoA-G2 | CGAGCCGAATATGGTCAT |
| hmoB-U1 | CACCGCCTGTCTTGTTAT |
| hmoB-U2 | TTCATTTCAGCCACCTCCT |
| hmoB-D1q | AGGAGGTGGCTGAAATGAAAACAAGACAACTCGCTGTA |
| hmoB-D2 | AAGATGAGTGCCGAATATCA |
| hmoB-CR1q | TGATATTCGGCACTCATCTTACTTCAACTAAAGCACCCAT |
| hmoB-CR2 | GTCTTCTTCCACCACTTG |
| hmoB-G1q | ATCAAGTGGTGGAAGAAGACGAGGATTAAGGCATCATCTGA |
| hmoB-G2 | GCAGCAGTTGTTGTTGTC |
| **Gene overexpression** | |
| ywfM-hemA-P1q | TCTTACATACAGATAACGACGACATACAGCCATTGAACATACG |
| ywfM-hemA-P2 | GTGTAAATTCCTCTCTTACCTAT |
| ywfM-hemA-1q | TATAGGTAAGAGAGGAATTTACACATGCATATACTTGTTGTGGGAG |
| ywfM-hemA-2 | CTCATTAAGACTTGCCATTGC |
| ywfM-hemA-T1q | GCAATGGCAAGTCTTAATGAGAAGCGAAATCCCGAGTCAA |
| ywfM-hemA-T2 | CTGGAAGTAGCACCTTACAA |
| ywfM-hemA-D1q | TTTGTAAGGTGCTACTTCCAGGGCAGAACACGAGTCTTA |
| ywfM-hemA-G2 | CGCCGCAGAAATGACCAA |
| CX-P*_43_*-1 | ATACAGCCATTGAACATACG |
| rocG-RcALAS-U2q | CCGTATGTTCAATGGCTGTATAGTAGGCTGTGGACACTT |
| rocG-RcALAS-1q | TATAGGTAAGAGAGGAATTTACACATGGATTACAACCTTGCTCT |
| rocG-RcALAS-2 | CGGATAAGAATTTCACACAG |
| rocG-RcALAS-T1q | CCTGTGTGAAATTCTTATCCGAAGCGAAATCCCGAGTCAA |
| rocG-RcALAS-T2 | GAAGTAGCACCTTACAAACTAT |
| rocG-RcALAS-D1q | CATAGTTTGTAAGGTGCTACTTCTCGGATGGGTCTAATTTGA |
| rocG-RpALAS-1q | TATAGGTAAGAGAGGAATTTACACATGAACTACGAAGCTTACTT |
| rocG-BjALAS-1q | TATAGGTAAGAGAGGAATTTACACATGGATTACGCTCAATTCTTC |
| rocG-AtALAS-1q | TATAGGTAAGAGAGGAATTTACACATGGATTTCGAAGCTTTCTTC |
| gcvTP-hemCDB-U2 | GTATAGCCAGTGCGGGAA |
| gcvTP-hemCDB-P1q | TTTCCCGCACTGGCTATACGACACGCAAATGATGAATACT |
| gcvTP-hemCDB-P2 | TTTTCCTCTCTCCCCTCTAAT |
| gcvTP-hemCDB-1q | ATTAGAGGGGAGAGAGGAAAAATGATGAGAACGATTAAAGTAGGTT |
| gcvTP-hemCDB-2 | ATTACTCCGCAAGCCATTTC |
| gcvTP-hemCDB-T1q | GAAATGGCTTGCGGAGTAATATGCGAAATCACGAGTCAA |
| gcvTP-hemCDB-T2 | CTGGAAGTAGAACCTTACAA |
| gcvTP-hemCDB-D1q | TTTGTAAGGTTCTACTTCCAGTGCCATATTGCGATCTTCC |
| gcvTP-hemCDB-G2 | TGCCAGGTGCTGATTACT |
| CX-hemCDB-1 | AGAGTCAGGAGCATTTAACC |
| CX-hemCDB-2 | CGGAATCGTTTCGTAGAGA |
| CX-hemCDB-3 | CGTATCGGTTGATGTGATG |
| CX-hemCDB-4 | GGCTTCATAGGTCCAGTC |
| ywjI-U1 | GGCTTCCAGAGATTTCAGA |
| ywjI-ccm-U2q | GAGTATTCATCATTTGCGTGTCTCATACCTACCAATTCCATT |
| ywjI-ccm-P1 | GACACGCAAATGATGAATACT |
| ywjI-ccm-1q | GATTAGAGGGGAGAGAGGAAAAATGGGTATGCTAGAAGCCAGA |
| ywjI-ccm-2 | AACCACCAACGGAATAAC |
| ywjI-ccm-D1q | CGTTATTCCGTTGGTGGTTTACAGTTCGTTTCGTTGAC |
| ywjI-G2 | TTGTGACAGCGAGTGATT |
| CX-ccm-2 | CTGCCACTGCCATTGATG |
| **RT-qPCR** | |
| RT-ccpA-1 | ACGAGCATGTGGCGGAATT |
| RT-ccpA-2 | CGATAGCGACTGACGGTGTT |
| RT-hemA-1 | GCAAGGTCACTGTCATTAAC |
| RT-hemA-2 | AATATCAGCCTCCGCAAG |
| RT-hemD-1 | TGCCACAGGAGTATATTGC |
| RT-hemD-2 | TTCATCACGGTAATGGTCTC |
| RT-hemH-1 | CAGCGTTCAGTCCTACAA |
| RT-hemH-2 | TTCCTTCACTCGGTCAAC |
| RT-hemQ-1 | TTGCTGAGTTCACCATTCC |
| RT-hemQ-2 | CAGTTGTCGTTGCCAGAT |

**Supporting Figures & Figure Captions**


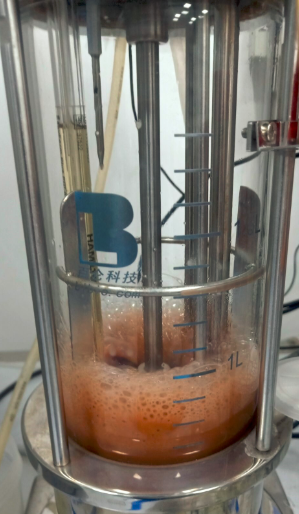

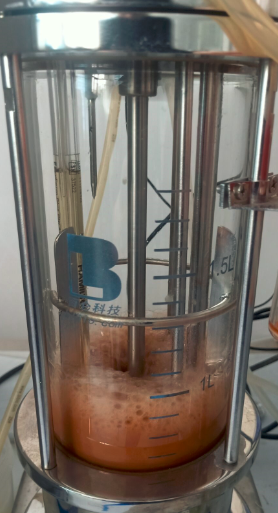

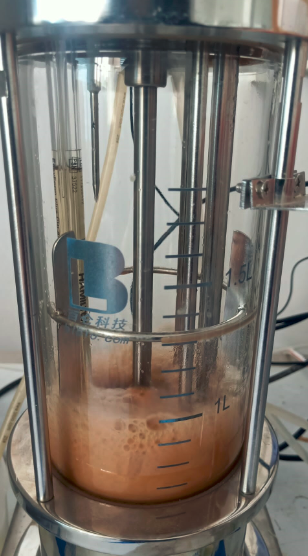

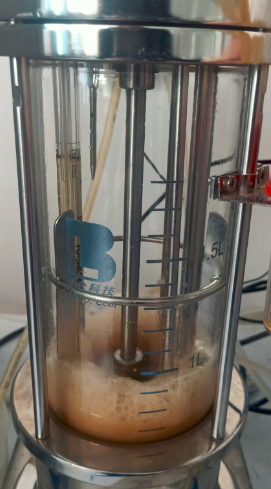

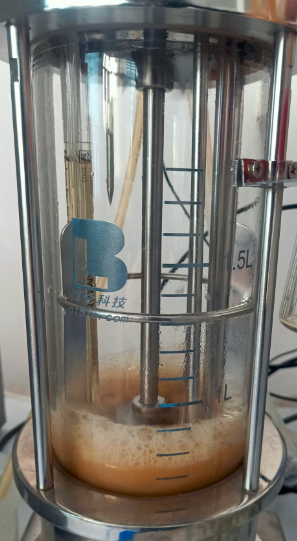

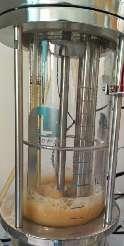

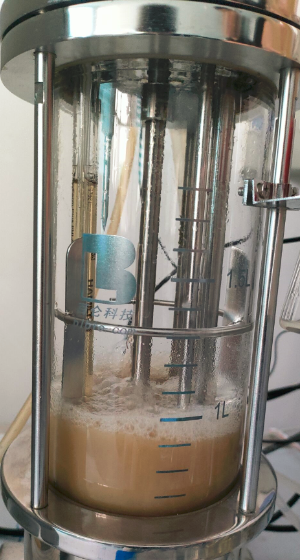

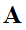

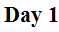

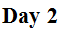

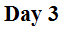

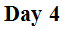

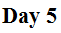

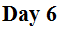

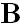

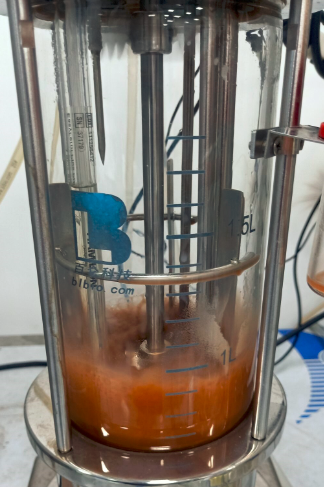

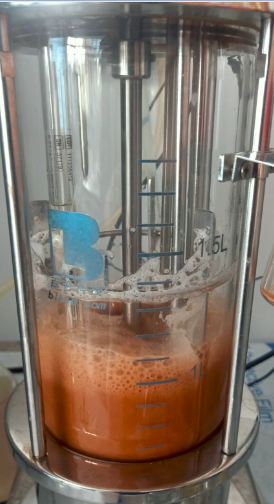

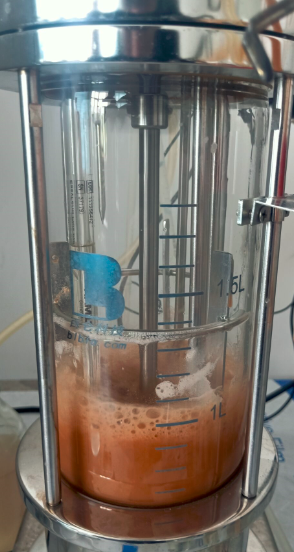

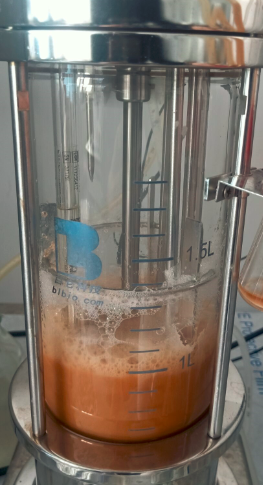

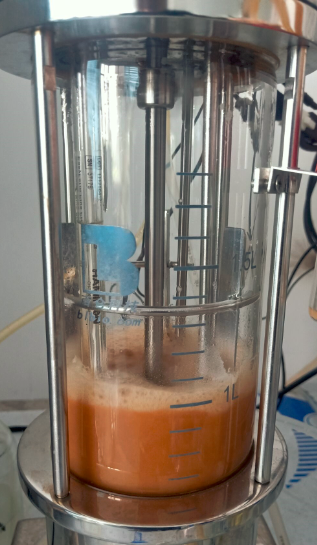

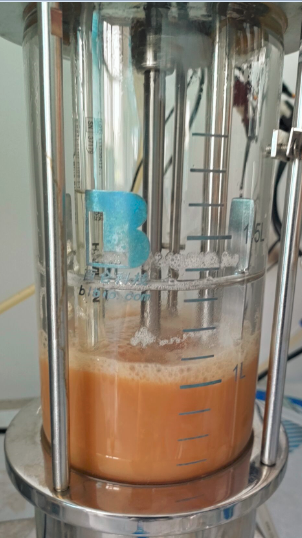

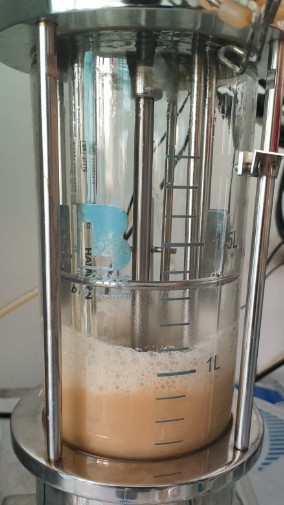

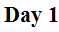

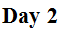

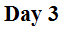

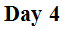

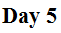

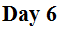

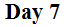

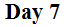


**Fig. S1** Color changes of the fermentation medium during fed-batch fermentation in a 2 L fermenter: (A) BSH11 fermented with sucrose as carbon source in both the fermentation medium and the feed solution for 7 days. (B) BSH11 fermented with glucose as carbon source for 7 days.

**Supporting Methods**

Construction of *ywfM* knockout strain. Fragments of 1,245 bp U_ywfM_, 1,197 bp D_ywfM_, and 691 bp G_ywfM_ were amplified from the *B. subtilis* 168 genome using the primer pairs ywfM-U1/ywfM-U2, ywfM-D1q/ywfM-D2, and ywfM-G1q/ywfM-G2, respectively. The 2,068 bp CR fragment was amplified from BS168NUm using the primer pair ywfM-CR1q/ywfM-CR2. These four PCR fragments were then ligated in the order of U-D-CR-G by splicing with two rounds of OE-PCR using the primer pairs ywfM-U1/ywfM-D2 and ywfM-U1/ywfM-G2, respectively. Finally, the UDCRG fragment was transformed into the competent cells of BSH1. Bacteria Liquid PCR was performed using the primer pair ywfM-U1/ywfM-D2. After the agarose gel electrophoresis, the size of PCR product was determined as 2,442 bp, and the transformant was correct. Otherswise, if the fragment size was 3,367 bp, then the transfromant was incorrect. The recombinant strain BSH2 was obtained after the CR was ejected.

Construction of *rocG* knockout strain. Fragments of 1,288 bp U_rocG_, 992 bp D_rocG_, and 938 bp G_rocG_ were amplified from the *B. subtilis* 168 genome using the primer pairs rocG-U1/rocG-U2q, rocG-D1/rocG-D2, and rocG-G1q/rocG-G2, respectively. The 2,155 bp CR fragment was amplified from BS168NUm using the primer pair rocG-CR1q/rocG-CR2. These four PCR fragments were then ligated in the order of U-D-CR-G by splicing with two rounds of OE-PCR using the primer pairs rocG-U1/rocG-D2 and rocG-U1/rocG-G2, respectively. Finally, the UDCRG fragment was transformed into the competent cells of BSH3. Bacteria Liquid PCR was performed using the primer pair rocG-U1/rocG-D2. After the agarose gel electrophoresis, the size of PCR product was determined as 2,280 bp, and the transformant was correct. Otherwise, if the fragment size was 3,336 bp, then the transformant was incorrect. The recombinant strain BSH4 was obtained after the CR was ejected.

Construction of *gcvTP* (*gcvT-gcvPA-gcvPB*) knockout strain. Fragments of 1,474 bp U_gcvTP_, 967 bp D_gcvTP_, and 1,464 bp G_gcvTP_ were amplified from the BS168N genome using the primer pairs gcvTP-U1/gcvTP-U2, gcvTP-D1q/gcvTP-D2, and gcvTP-G1q/gcvTP-G2, respectively. The 2,138 bp CR fragment was amplified from BS168NUm using the primer pair gcvTP-CR1q/gcvTP-CR2. These four PCR fragments were then ligated in the order of U-D-CR-G by splicing with two rounds of OE-PCR using the primer pairs gcvTP-U1/gcvTP-D2 and gcvTP-U1/gcvTP-G2, respectively. Finally, the UDCRG fragment was transformed into the competent cells of BSH53. Bacteria Liquid PCR was performed using the primer pair gcvTP-U1/gcvTP-D2. After the agarose gel electrophoresis, the size of PCR product was determined as 2,441 bp, and the transformant was correct. Otherwise, if the fragment size was 5,310 bp, then the transformant was incorrect. The recombinant strain BSH6 was obtained after the CR was ejected.

Construction of *hemCDB* (*hemC-hemD-hemB*) overexpression strain. Fragments of 1,474 bp U_hemCDB_, 442 bp P_hemCDB_ (containing the sequence of the constitutive promoter P*_lapS_*), 2,695 bp CDB_hemCDB_ (containing the gene sequence of *hemCDB*), and 259 bp T_hemA_ (containing the terminator sequence of *pyrE*) were amplified from the *B. subtilis* 168 genome using the primer pairs gcvTP-U1/gcvTP-hemCDB-U2, gcvTP-hemCDB-P1q/gcvTP-hemCDB-P2, gcvTP-hemCDB-1q/gcvTP-hemCDB-2, and gcvTP-hemCDB-T1q/gcvTP-hemCDB-T2, respectively. The 4,448 bp DCRG fragment was amplified from BSH53m (BSH53, Δ*gcvTP*::D-*cat-araR*) using the primer pair gcvTP-hemCDB-D1q/gcvTP-hemCDB-G2. These five PCR fragments were then ligated in the order of U-P-CDB-T-DCRG by splicing with two rounds of OE-PCR using the primer pairs gcvTP-hemCDB-P1q/gcvTP-hemCDB-T2 and gcvTP-U1/gcvTP-hemCDB-G2, respectively. Finally, the competent cells of BSH53 were transformed using the UPCDBTDCRG fragment yielding strain BSH7. The successful transformation was verified by a two-step screening process. DNA sequencing was performed by using the primers CX-hemCDB-1, CX-hemCDB-2, CX-hemCDB-3, and CX-hemCDB-4.

Construction of *ccmABC* (*ccmA-ccmB-ccmC*) overexpression strain. Fragments of 1,230 bp U_ccm_ and 442 bp P_ccm_ (containing the sequence of the constitutive promoter P*_lapS_*) were amplified from the *B. subtilis* 168 genome using the primer pairs ywjI-U1/ywjI-ccm-U2q and ywjI-ccm-P1/gcvTP-hemCDB-P2, respectively. The 2,160 bp ABC_ccm_ fragment (containing the gene sequence of *ccmABC*) was amplified from the *E. coli* DH5α genome using the primer pair ywjI-ccm-1q/ywjI-ccm-2. The 3,756 bp DCRG fragment, with fragment D containing the terminator sequence, was amplified from BS168NYm (BS168N, Δ*ywjI*::D-*cat-araR*) using the primer pair ywjI-ccm-D1q/ywjI-G2. These four PCR fragments were then ligated in the order of U-P-ABC-DCRG by splicing with two rounds of OE-PCR using the primer pairs ywjI-ccm-P1/ywjI-ccm-2 and ywjI-U1/ywjI-G2, respectively. Finally, the competent cells of BSH7 were transformed using the UPABCDCRG fragment yielding strain BSH8. The successful transformation was verified by a two-step screening process. DNA sequencing was performed by using the primers CX-hemCDB-1, CX-ccm-2, and ywjI-ccm-2.

Construction of *nasF* knockout strain. Fragments of 1,155 bp U_nasF_, 1,487 bp D_nasF_, and 990 bp G_nasF_ were amplified from the BS168N genome using the primer pairs nasF-U1/nasF-U2, nasF-D1q/nasF-D2, and nasF-G1q/nasF-G2, respectively. The 2,136 bp CR fragment was amplified from BS168NUm using the primer pair nasF-CR1q/hemX-CR2. These four PCR fragments were then ligated in the order of U-D-CR-G by splicing with two rounds of OE-PCR using the primer pairs nasF-U1/nasF-D2 and nasF-U1/nasF-G2, respectively. Finally, the UDCRG fragment was transformed into the competent cells of BSH8. Bacteria Liquid PCR was performed using the primer pair nasF-U1/nasF-D2. After the agarose gel electrophoresis, the size of PCR product was determined as 2,642 bp, and the transformant was correct. Otherwise, if the fragment size was 3,977 bp, then the transformant was incorrect. The recombinant strain BSH9 was obtained after the CR was ejected.

Construction of *hmoA* (*yetG*) knockout strain. Fragments of 1,254 bp U_hmoA_, 1,184 bp D_hmoA_, and 646 bp G_hmoA_ were amplified from the BS168N genome using the primer pairs hmoA-U1/hmoA-U2, hmoA-D1q/hmoA-D2, and hmoA-G1q/hmoA-G2, respectively. The 2,136 bp CR fragment was amplified from BS168NUm using the primer pair hmoA-CR1q/rocG-CR2. These four PCR fragments were then ligated in the order of U-D-CR-G by splicing with two rounds of OE-PCR using the primer pairs hmoA-U1/hmoA-D2 and hmoA-U1/hmoA-G2, respectively. Finally, the UDCRG fragment was used to transform into the competent cells of BSH9. Bacteria Liquid PCR was performed using the primer pair hmoA-U1/hmoA-D2. After the agarose gel electrophoresis, the size of PCR product was determined as 2,438 bp, and the transformant was correct. Otherwise, if the fragment size was 3,467 bp, then the transformant was incorrect. The recombinant strain BSH10 was obtained after the CR was ejected.

Construction of *hmoB* (*yhgC*) knockout strain. Fragments of 1,126 bp U_hmoB_, 1,289 bp D_hmoB_, and 741 bp G_hmoB_ were amplified from the BS168N genome, using the primer pairs hmoB-U1/hmoB-U2, hmoB-D1q/hmoB-D2, and hmoB-G1q/hmoB-G2, respectively. The 2,089 bp CR fragment was amplified from BS168NUm using the primer pair hmoB-CR1q/hmoB-CR2. These four PCR fragments were then ligated in the order of U-D-CR-G by splicing with two rounds of OE-PCR using the primer pairs hmoB-U1/hmoB-D2 and hmoB-U1/hmoB-G2, respectively. Finally, the UDCRG fragment was transformed into the competent cells of BSH10. Bacteria Liquid PCR was performed using the primer pair hmoB-U1/hmoB-D2. After the agarose gel electrophoresis, the size of PCR product was determined as 2,415 bp, and the transformant was correct. Otherwise, if the fragment size was 4,020 bp, then the transformant was incorrect. The recombinant strain BSH11 was obtained after the CR was ejected.
